# A scoring model for diagnosis of tuberculous pleural effusion

**DOI:** 10.1186/s12890-022-02131-7

**Published:** 2022-09-02

**Authors:** Senquan Wu, Shaomei Li, Nianxin Fang, Weiliang Mo, Huadong Wang, Ping Zhang

**Affiliations:** 1 Department of Respiratory and Critical Care Medicine, Dongguan People’s Hospital, 78 Wandao Road South, Dongguan, 523059 Guangdong China; 2 Department of Pathophysiology, Key Laboratory of State Administration of Traditional Chinese Medicine of the People’s Republic of China, School of Medicine, Jinan University, Guangzhou, 510632 Guangdong China

**Keywords:** Tuberculosis, Pleural effusion, Tuberculous pleural effusion, Scoring model

## Abstract

**Background:**

Due to the low efficiency of a single clinical feature or laboratory variable in the diagnosis of tuberculous pleural effusion (TBPE), the diagnosis of TBPE is still challenging. This study aimed to build a scoring diagnostic model based on laboratory variables and clinical features to differentiate TBPE from non-tuberculous pleural effusion (non-TBPE).

**Methods:**

A retrospective study of 125 patients (63 with TBPE; 62 with non-TBPE) was undertaken. Univariate analysis was used to select the laboratory and clinical variables relevant to the model composition. The statistically different variables were selected to undergo binary logistic regression. Variables B coefficients were used to define a numerical score to calculate a scoring model. A receiver operating characteristic (ROC) curve was used to calculate the best cut-off value and evaluate the performance of the model. Finally, we add a validation cohort to verify the model.

**Results:**

Six variables were selected in the scoring model: Age ≤ 46 years old (4.96 points), Male (2.44 points), No cancer (3.19 points), Positive T-cell Spot (T-SPOT) results (4.69 points), Adenosine Deaminase (ADA) ≥ 24.5U/L (2.48 point), C-reactive Protein (CRP) ≥ 52.8 mg/L (1.84 points). With a cut-off value of a total score of 11.038 points, the scoring model’s sensitivity, specificity, and accuracy were 93.7%, 96.8%, and 99.2%, respectively. And the validation cohort confirms the model with the sensitivity, specificity, and accuracy of 92.9%, 93.3%, and 93.1%, respectively.

**Conclusion:**

The scoring model can be used in differentiating TBPE from non-TBPE.

## Introduction

Tuberculosis (TB) affects about nine million people and causes 1.5 million deaths worldwide every year [1]. In 2016, tuberculosis claimed 1.3 million lives among HIV-negative people, exceeding the total number of deaths caused by HIV and becoming the first killer among infectious diseases [2]. On Sep 26, 2018, all UN Member States promised to end the global tuberculosis epidemic by 2030 in the UN General Assembly High Level meeting [3]. Currently, pleural tuberculosis (PT) is the most common type of extrapulmonary tuberculosis, and the frequency of all TB varies significantly in different countries. PT accounts for more than 20% of all TB cases in Africa [4, 5], 6.5 to 8.7% in China [6, 7], 8.7% in Brazil [8], and 3.7% in the United States [9]. PT appears to be the leading cause of pleural effusion (PE) in countries with a high prevalence of tuberculosis (e.g., in India) [10].

Tuberculous pleural effusion (TBPE) accounts for about 40% of PE cases in China [11, 12]. For patients with TBPE, untimely anti-tuberculosis treatment will affect its prognosis. The diagnostic criteria of TBPE is dependent on bacteria culture and histopathology, but the diagnostic sensitivity is limited and time-consuming. Thoracoscopic Pleural Biopsy is an effective method in diagnosing TBPE, but its application is limited because of its invasiveness, complexity, and technical difficulty [5, 13, 14]. Moreover, the diagnostic value of a single clinical biomarker of TBPE is limited, including erythrocyte sedimentation rate (ESR), blood T-cell spot (T-SPOT), adenosine deaminase (ADA), and lymphocyte ratio, so the diagnosis of TBPE is still challenging. Thus, our study aimed to build a scoring diagnostic model based on laboratory variables and clinical features to differentiate TBPE from non-TBPE.

## Methods

### Study subjects

A retrospective study was conducted from 2016 to 2021 after approval by the Ethics Committee of Dongguan People's Hospital. All the enrolled patients met the indications of thoracic puncture or thoracoscopic pleural biopsy and signed the informed contents. The patients selected in the study should meet the following criteria: (1) Adult group; (2) Presence of PE on chest radiographs and ultrasonography; (3) Complete data in clinical were available for all patients. Finally, 125 PE patients were included in the retrospective study. In addition, we collected data from 29 new patients diagnosed with TBPE or non-TBPE to validate the diagnostic model. The data we collected mainly included six factors in the diagnostic model in calculating the model's score.

### Diagnostic criteria

TBPE diagnosis was confirmed when PE met at least one of the following criteria: (1) pleural fluid/sputum/bronchial aspirate/bronchoscopic brushing specimen was positive for acid-fast bacilli or positive culture or positive polymerase chain reaction (PCR) for Mycobacterium tuberculosis. (2) Positive acid-fast staining or epithelioid caseous granuloma in pleural or lung tissue [15–17].

Malignant Pleural Effusion (MPE) was diagnosed when pleural histopathology demonstrated malignant lesions or when cytological examination of pleural effusion demonstrated malignant cells.

Empyema cases with negative M. tuberculosis culture were confirmed according to ATS guidelines [18]. Parapneumonic effusion (PPE) was caused by pneumonia which was approved based on the criteria of the American Thoracic Society (ATS) [19]. Thoracoscopic pleural biopsy was performed in patients with unknown etiology of PE. Except for the cases with TBPE, all the other cases were classified as a non-TBPE group.

All patients underwent a standard thoracocentesis procedure to collect pleural effusion samples, and blood was collected by venepuncture before intervention procedures. Record items include sex, age, clinical symptoms (cough, fever, chest pain, night sweats), T-SPOT, ESR, C-reactive protein (CRP), PE lymphocyte ratio, PE protein, PE lactate dehydrogenase (LDH), ADA, PE location, presence of cancer or not. Patients who had incomplete data were excluded from this study. The statistical analysis was performed using the first pleural fluid sample data before treatment. Hematological data were collected from the blood samples nearest to the first thoracentesis.

T-SPOT TB test was measured using Enzyme-Linked Immunospot (T-SPOT TB assay kit, Oxford Immunotec Co., Ltd., Abingdon, UK). Pleural effusion protein was measured using Colorimetric Determination (Dry tablets assay kit, Ortho-Clinical Diagnostics Co., Ltd., New York, USA). The activity of ADA was measured using a peroxidase assay (ADA assay kit, Beijing Leadman Biochemistry Co., Ltd., Beijing, China). LDH levels were measured using the lactic acid substrate method (LDH assay kit, Beckman Coulter Laboratory Systems Co., Ltd., Suzhou, China). CRP level was measured using the Quantitative Immunofluorescence method (Boditech Biotechnology Co., Ltd., Guangxi, China). ESR was measured using an Italian.

Microsed-System Automatic Blood Sedimentation Instrument. Differential cell counts in PE were counted manually.

### Statistical analysis

The analyses were carried out using SPSS 22.0 software (SPSS Inc., Chicago, IL, USA). Continuous data are reported as median, with first and third quartiles. Mann–Whitney U test was used for comparisons between groups. The Chi-square test was used for the analysis of categorical variables. The results with *p* values less than 0.05 were considered statistical significance. Sensitivity, specificity, positive predictive value (PPV), negative predictive value (NPV), accuracy, and the Youden index were calculated to estimate the diagnostic performance of the indicators. In addition, the receiver operating characteristic (ROC) curve was plotted to evaluate the diagnostic value of continuous data for TBPE. The continuous variable was converted into a binary variable according to the cut-off value corresponding to the maximum value of the Youden index. The factors with statistically significant differences between the two groups were selected in the binary logistic regression. A goodness-of-fit test of the binary logistic regression model was evaluated by Hosmer and Lemeshow test. Variables B coefficients were used to define a numerical score to calculate a scoring model. A receiver operating characteristic (ROC) curve was used to calculate the best cut-off value and evaluate the performance of the scoring model. Finally, a statistical evaluation of the diagnostic scoring model was performed in a validation cohort.

## Results

### Clinical and laboratory findings of the 125 patients with PE

In this study, 125 patients (63 with TBPE; 62 with non-TBPE) were enrolled according to the selection criteria. The demographic and clinical characteristics were collected from the patient's medical records and summarized in Table 1. There was no significant difference between the two groups in cough, chest pain, night sweats, pleural fluid protein, and LDH (*p* > 0.05). Still, there were significant differences among age, male, cancer diagnosis, pleural fluid location, fever, ESR, CRP, T-SPOT, pleural fluid lymphocyte ratio, and ADA (*p* < 0.05). The mean age of the TBPE group was lower than that of the non-TBPE group. TBPE predominated in men (46/63, 73.0%) who with unilateral pleural effusion (62/63, 98.4%). MPE accounts for most of the non-TBPE (43/62, 69.4%). The proportion of fever, positive T-SPOT, and the mean values of ESR, CRP, pleural fluid lymphocyte ratio, and ADA in patients with TBPE was higher than in patients with non-TBPE.

**Table 1:** Clinical and laboratory findings of the 125 patients with PE

	Total (n = 125)	TBPE (n = 63)	Non-TBPE (n = 62)	*p* value
Age (years)	58.0 (38.0–70.5)	42.0 (29.0–65.0)	67.0 (53.0–74.0)	0.000
Male	71 (56.8%)	46 (73.0%)	25 (40.3%)	0.000
Cancer	44 (35.2%)	1 (1.6%)	43 (69.4%)	0.000
Unilateral PE	116 (92.8%)	62 (98.4%)	54 (87.1%)	0.017
Cough	101 (80.8%)	52 (82.5%)	49 (79.0%)	0.656
Fever	41 (32.8%)	33 (52.4%)	8 (12.9%)	0.000
Chest pain	50 (40.0%)	30 (47.6%)	20 (32.3%)	0.080
Night sweats	4 (3.2%)	3 (4.8%)	1 (1.6%)	0.619
*In blood*				
ESR	35.0 (21.5–56.0)	40.0 (28.0–60.0)	30.0 (12.0–45.7)	0.031
CRP	36.1 (13.2–90.8)	76.5 (30.1–131.3)	18.1 (8.4–41.0)	0.000
T-SPOT	73 (58.4%)	59 (93.7%)	14 (22.6%)	0.000
*In PE*				
L%	90 (77.5–96.0)	93.0 (84.0–98.0)	85.0 (67.7–94.0)	0.000
Protein	64.7 (53.0–79.6)	69.5 (58.5–81.6)	61.7 (50.4–77.5)	0.063
ADA	26.7 (8.6–41.7)	40.9 (33.6–49.3)	9.5 (7.1–12.9)	0.000
LDH	306.2 (216.2–571.4)	415.1 (262.0–610.7)	269.4 (207.7–392.9)	0.299

Data in the table are expressed either as a frequency (percentage) or a median (interquartile range)

*PE* Pleural effusion, *TBPE* Tuberculous pleural effusion, *Non-TBPE* Non-tuberculous pleural effusion, *ESR* Erythrocyte sedimentation rate, *CRP* C-reactive protein, *T-SPOT* T-cell spot, *L*% Lymphocyte ratio, *ADA* Adenosine deaminase

### The diagnostic performance of a single indicator for TBPE

**Fig. 1:**
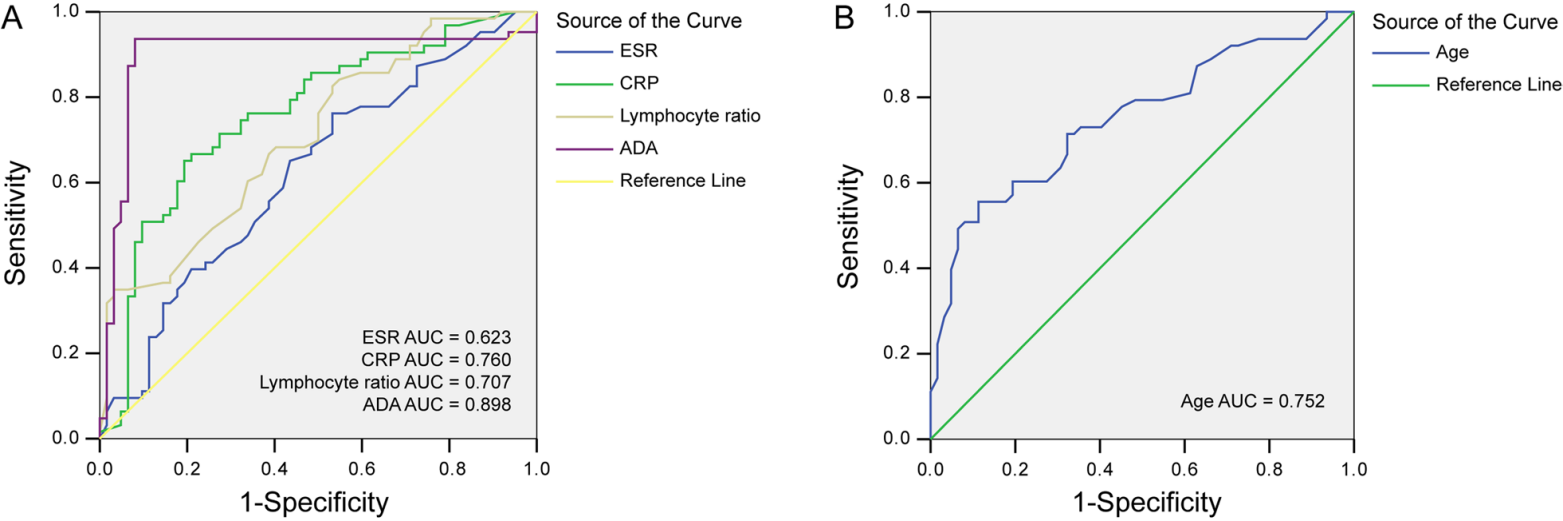
The diagnostic value for TBPE. **A** The diagnostic value of ESR, CRP, Lymphocyte ratio, and ADA for TBPE, the AUC value was 0.623, 0.760, 0.707, and 0.898, respectively; AUC: area under the curve. **B** The diagnostic value of Age for TBPE, the AUC value, was 0.752

ROC curve was used to evaluate the diagnostic value of continuous data (Fig. 1A, B). The continuous variable was converted into a binary variable according to the cut-off value corresponding to the maximum value of the Youden Index (Table 2). The diagnostic performance, including Sensitivity, Specificity, Positive predictive value (PPV), Negative predictive value (NPV), and Accuracy, were calculated and summarized in Table 3.

**Table 2:** Youden index and the cut-off value of continuous data

	Youden index	Cut-off value
Age	0.443	46.5
ESR	0.23	27.5
CRP	0.457	52.8
L%	0.264	91.5
ADA	0.856	24.5

*ESR* Erythrocyte sedimentation rate, *CRP* C-reactive protein, *L*% Lymphocyte ratio, *ADA* Adenosine deaminase

**Table 3:** The diagnostic performance of a single indicator for TBPE

	Sensitivity	Specificity	PPV	NPV	Accuracy
Age ≤ 46.5 years	0.556	0.887	0.833	0.663	0.72
Male	0.73	0.597	0.648	0.685	0.664
No cancer	0.984	0.694	0.765	0.977	0.84
Fever	0.524	0.871	0.805	0.643	0.696
Unilateral PE	0.984	0.129	0.534	0.889	0.56
Positive T-SPOT	0.937	0.774	0.808	0.923	0.856
ESR ≥ 27.5 mm/h	0.762	0.468	0.593	0.659	0.616
CRP ≥ 52.8 mg/L	0.651	0.806	0.774	0.694	0.728
L% ≥ 91.5%	0.603	0.661	0.644	0.621	0.632
ADA ≥ 24.5 U/L	0.937	0.919	0.922	0.934	0.928

*PPV* Positive predictive value, *NPV* Negative predictive value, *PE* Pleural effusion, *T-SPOT* T-cell spot, *ESR* Erythrocyte sedimentation rate, *CRP* C-reactive protein, *L*% Lymphocyte ratio, *ADA* Adenosine deaminase

### The multivariate logistic regression of binary variables and the establishment of the scoring model

The factors with statistically significant differences between the two groups were selected in the logistic regression, using the forward selection method to select indicators to enter the final model. At last, ESR ≥ 27.5 mm/h, fever, lymphocyte ratio ≥ 91.5%, unilateral pleural effusion was eliminated by the model, And age ≤ 46.5 years, male, no cancer, positive T-SPOT, CRP ≥ 52.8 mg/L, ADA ≥ 24.5 U/L were accepted into the final binary logistic regression model. Hosmer and Lemeshow test Confirmed a good goodness-of-fit test of the binary logistic regression model (*p* = 0.499). Variables B coefficients were used to define a numerical score to calculate a scoring model (Table 4).

**Table 4:** Score for diagnosis based on the B coefficient of the variables

Variable	Scoring criteria	B coefficient	Score
Age	Age ≤ 46.5 years	4.96	4.96
Gender	Male	2.44	2.44
Cancer	No cancer	3.19	3.19
T-SPOT	Positive T-SPOT	4.69	4.69
CRP	CRP ≥ 52.8 mg/L	1.84	1.84
ADA	ADA ≥ 24.5U/L	2.48	2.48

*T-SPOT* T-cell spot, *CRP* C-reactive protein, *ADA* Adenosine deaminase

### The diagnostic performance of the scoring model

**Fig. 2:**
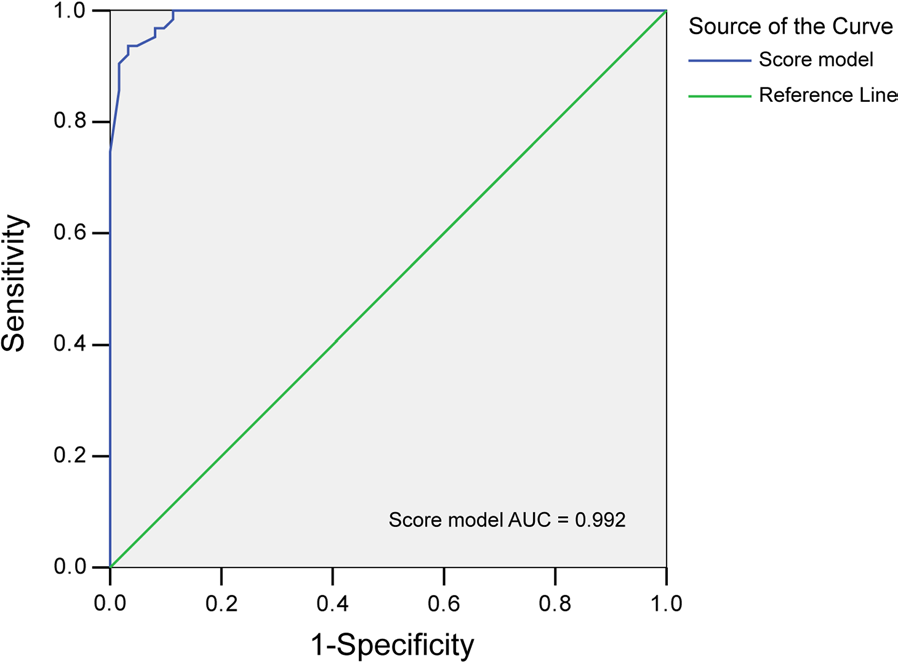
The diagnostic value of the Scoring model for TBPE. The AUC value was 0.992; *AUC* Area under the curve

The total score is equal to the sum Variables score in Table 4 when matching the scoring criteria. The calculation equation as follow: Y (total score) = Age score + Gender score + Cancer score + T-SPOT score + CRP score + ADA score. ROC curve was used to calculate the best cut-off value. When the total score was ≥ 11.038, The area under the curve (AUC) value was 0.992 (95%CI 0.982–1.000) (Fig. 2). The scoring model's performance for diagnosing TBPE was summarized in Table 5.

**Table 5:** The performance of the scoring model

	Scoring model
Cut-off value	≥ 11.038
Youden index	0.905
Sensitivity	0.937
Specificity	0.968
Accuracy	0.992
PPV	1.000
NPV	0.939

*PPV* Positive predictive value, *NPV* Negative predictive value

### Validation of the diagnostic scoring model

Twenty-nine new patients with PE in the validation cohort were from the same retrospective database from 2016–2021 and approved by the Ethics Committee of Dongguan People’s Hospital, of which 14 were diagnosed with TBPE; among the remaining 15 patients were non-TBPE; 13 of 14 patients with TBPE had a score of ≥ 11.038; 1 of 15 patients with non-TBPE had a score of ≥ 11.038. The baseline characteristics with six indicators included in the model in the validation cohort were summarized in Table 6. And the validation results of the scoring model are shown in Table 7.

**Table 6:** The baseline characteristics of the validation cohort

	Total (n = 29)	TBPE (14)	Non-TBPE (n = 15)	*p* value
Age (years)	49.00 (35.5–75.5)	37.50 (28.50–48.25)	75.00 (70.00–82.00)	0.000
Male	16 (55.17%)	12 (85.71%)	4 (26.67%)	0.003
Cancer	10 (34.48%)	0 (0%)	10 (66.67%)	0.000
T-SPOT	15 (51.72%)	13 (92.85%)	2 (13.33%)	0.000
CRP (mg/L)	40.50 (18.56–74.66)	74.63 (40.38–129.14)	22.97 (6.32–54.76)	0.002
ADA (U/L)	26.2 (9.70–41.35)	41.35 (32.62–63.15)	10.00 (5.70–25.00)	0.000

Data in the table are expressed either as a frequency (percentage) or a median (interquartile range)

*TBPE* Tuberculous pleural effusion, *Non-TBPE* Non-tuberculous pleural effusion, *T-SPOT* T-cell spot, *CRP* C-reactive protein, *ADA* Adenosine deaminase

**Table 7:** The validation results of the scoring model

Diagnostic index	Scoring model
Sensitivity	0.929
Specificity	0.933
Accuracy	0.931
PPV	0.929
NPV	0.933

*PPV* Positive predictive value, *NPV* Negative predictive value

## Discussion

There are many causes of pleural effusion. Since the high prevalence of TB occurred in China, TBPE accounts for about 40% of pleural effusion [11, 12]. Currently, the diagnostic performance of clinical features or laboratory variables in diagnosing TBPE is poor; therefore, it’s an urgent need to find a new method to diagnose TBPE. Our study built a scoring diagnostic model by using logistic regression based on laboratory variables and clinical features to differentiate TBPE from non-TBPE.

A total of 15 indicators were included in this study. No symptoms were introduced into the final binary logistic regression model, which shows that clinical symptoms had no significant diagnosis value to the TBPE. And the results were similar to the previously reported data [20]. In terms of pleural effusion tests, unilateral pleural effusion, pleural effusion protein, LDH, and pleural effusion lymphocytes ratio ultimately failed to enter the final model, which may be due to TBPE and MPE being dominated by unilateral lymphocytic exudates [21]. Most of the non-TBPE in this study were MPE. As expected, ESR, a non-specific indicator for the diagnosis of TBPE, was eventually eliminated by the regression model, even though there was a difference in univariate analysis between the two groups.

Finally, six indicators of age, sex, cancer, CRP, T-SPOT, and ADA were included in the diagnostic scoring model by multivariate binary logistics regression, which showed that those six indicators significantly contributed significantly to the diagnosis. In terms of age, the non-TBPE group was significantly older than the TBPE group, which may attribute to the elderly-onset of cancer that was the leading cause of non-TBPE. Our result showed that TBPE predominated in men (46/63; 73.0%), and it was similar to the previously reported data of Roberta et al. [21]. Consistent with the previous studies [22], we also demonstrated the potential diagnostic value of CRP for TBPE. The sensitivity and specificity of ADA, which had an excellent diagnostic performance in diagnosing TBPE, were above 90% in diagnosing TBPE. T-SPOT also showed an excellent diagnostic value for the diagnosis of TBPE that the sensitivity and specificity were 93.7% and 77.4%, respectively. The above results were similar to the previous studies [20, 23, 24].

Hosmer and Lemeshow test confirmed a good goodness-of-fit test of the binary logistic regression model (*p* = 0.499). The performance of the scoring model for diagnosis of TBPE was evaluated by the ROC curve; when the total score was ≥ 11.038, the AUC value was 0.992 (95%CI 0.982–1.000), the sensitivity, specificity, PPV, NPV were 93.7%, 96.8%, 100%, and 93.9% respectively, the diagnostic performance of the scoring model was better than the reported data of Petborom et al. [20, 25]. Our scoring model can aidin diagnosing TBPE and provide more evidence for anti-tuberculosis treatment. Still, we need to track the effectiveness of treatment to verify the accuracy of the diagnostic model follow-up. When anti-tuberculosis treatment is ineffective, a thoracoscopic pleural biopsy should be used to further confirm the diagnosis. For medical units with limited sanitary conditions, the diagnostic model can help to find TBPE, so that patients can be referred to specialist hospitals for earlier treatment.

The retrospective study has a selection bias; for example, patients with incomplete data were excluded from the study, leading to a reduction in the number of cases. Some test indicators, such as cytokines, CD4, and CD8, were not included in our study because of the small number of people tested. Therefore, establishing a better TBPE diagnostic scoring model requires a prospective, multi-center study to achieve.

## Conclusions

The diagnostic score model established by logistic regression combined with multiple indicators improves diagnostic performance. It is better than a single index of laboratory variables or clinical features in diagnosing TBPE. In brief, establishing a scoring model for diagnosing TBPE provides a good diagnostic method based on routine clinical data to assist clinicians in making better clinical decisions.

## Data Availability

Most of the data were included in the submission. More data details were available from the corresponding authors on reasonable request.

## Abbreviations

TB: Tuberculosis
TBPE: Tuberculous pleural effusion
ROC: Receiver operating characteristic
T-SPOT: T-cell spot
ADA: Adenosine deaminase
CRP: C-reactive protein
PT: Pleural tuberculosis
PE: Pleural effusion
ESR: Erythrocyte sedimentation rate
LDH: Lactate dehydrogenase
PPV: Positive predictive value
NPV: Negative predictive value
